# Changes in fatty acid levels (saturated, monounsaturated and polyunsaturated) during pregnancy

**DOI:** 10.1186/s12884-021-04251-0

**Published:** 2021-11-17

**Authors:** Estefania Aparicio, Carla Martín-Grau, Carmen Hernández-Martinez, Nuria Voltas, Josefa Canals, Victoria Arija

**Affiliations:** 1 Research Group on Nutrition and Mental Health (NUTRISAM), Universitat Rovira i Virgili, 43201 Reus, Spain; 2 Institut d’Investigació Sanitària Pere Virgili (IISPV), 43003 Tarragona, Spain; 3 Clinical Chemistry Laboratory, Catalan Institute of Health (ICS)-Camp de Tarragona-Terres de l’Ebre, Joan XXIII University Hospital in Tarragona, 43005 Tarragona, Spain; 4 Research Center for Behavior Assessment (CRAMC), Universitat Rovira i Virgili, Tarragona, Spain

**Keywords:** Pregnancy, Fatty acid status, DHA, EPA, Omega-3, Omega-6

## Abstract

**Background:**

During pregnancy a high amount of fatty acids (FA) is necessary to meet foetus demands, which vary during gestation. The present study describes the changes in maternal fatty acid concentrations during pregnancy in a sample of pregnant women.

**Methods:**

This is a longitudinal study of 479 pregnant women who were monitored from the first trimester to third trimester of pregnancy. Data on maternal characteristics were recorded and a serum sample was collected in each trimester. The fatty acid profile (saturated (SFA: total, lauric acid, myristic acid, palmitic acid, stearic acid), monounsaturated (MUFA: total, palmitoleic acid, oleic acid) and polyunsaturated fatty acids (PUFA: total omega-6 (n-6), linoleic acid, dihomo-γ-linolenic acid, arachidonic acid (AA), total omega-3 (n-3), eicosapentaenoic acid (EPA), docosahexaenoic acid (DHA)) was analysed with a gas chromatography-mass spectrometry combination.

**Results:**

From the first trimester to third trimester of pregnancy, a significant increase in total SFA, total MUFA and total n-6 PUFA was found. (*p* < 0.001). Nevertheless, the serum concentration of arachidonic acid (AA), eicosapentaenoic acid (EPA) and total n-3 PUFA decreased during gestation (*p* < 0.001). A statistically non-significant result was observed for the docosahexaenoic acid (DHA) serum concentration between the first and third trimesters of pregnancy. Significant correlations were observed between each total fatty acid concentrations of the first and third trimesters.

**Conclusion:**

The circulating serum concentration of SFA, MUFA and n-6 PUFA increases during pregnancy, whereas essential fatty acids such as AA and EPA decrease, and DHA remains unchanged. Further research is necessary to understand the role played by FA throughout gestation.

## Introduction

A good nutritional status of fatty acids (FA) during pregnancy is important for the mother’s health in order to meet the needs of foetal growth and development. Studies evaluating the effects of FA deficiency states during pregnancy have mainly focused on polyunsaturated fatty acids (PUFA). In particular, a maternal deficiency of omega-3 polyunsaturated fatty acids (n-3 PUFA) may affect placental angiogenesis and vasculogenesis, thereby affecting foetal development (brain, muscle, eye, motor nerve and adiposity) [1]. It is known that eicosapentaenoic acid (EPA) and docosahexaenoic acid (DHA) are two of the most important FA for the cognitive and visual development of the foetus [2–5]. Maternal fatty acid status is also crucial in late pregnancy because this is when the human foetal brain grows rapidly and high amounts of DHA accumulates in it [1, 5]. Thus, the foetus during the third trimester is especially vulnerable to developmental deficits due to a poor status of n-3 PUFA, and this could cause problems in neurological development [6, 7]. However, few studies have assessed the changes in FA during pregnancy [8–10]. An appropriate balance with n-3 and omega-6 polyunsaturated fatty acids (n-6 PUFA) is necessary [11, 12] because a high concentration of maternal arachidonic acid (AA) has been associated with deleterious outcomes, such as major depression in the mother [13, 14], or preterm delivery [15]. However, a higher maternal AA/DHA ratio at delivery and heterozygosity for the FADS1 genetic variant have shown positive long-term effects on processing speed in children at 9 years of age [7].

Therefore, an adequate fatty acid profile in early and late pregnancy is crucial for the health of both the mother and the child. To our knowledge there is a dearth of literature on the changes in plasma FA concentrations throughout pregnancy. A recent systematic review analysed how the n-3 and n-6 PUFA profile is modified during pregnancy [16]. It included only three studies [17–19] and showed that DHA and n-6 PUFA concentrations increase throughout pregnancy, while EPA remains unchanged. Similar findings were obtained in research conducted with a Canadian pregnant population with and without gestational diabetes [20]. Saturated fatty acids (SFA) and monounsaturated fatty acids (MUFA) have been studied less [17, 19, 21]. The survey by Pinto et al. [17] of 225 pregnant Brazilian women found an increase in SFA and MUFA concentrations throughout pregnancy. Taking into account the importance of FA status for maternal and foetal health, a better understanding and knowledge of this subject would help obstetrics and health professionals to identify possible abnormal values and decide whether treatment is necessary and which treatment to recommend. The aim of the current study is therefore to analyse the changes in maternal fatty acid concentrations (saturated, monounsaturated and polyunsaturated) during pregnancy in a cohort of pregnant women from a Mediterranean country in Europe.

## Material and methods

### Study design and population

This study researched a longitudinal cohort of pregnant women from the ECLIPSES study [22]. The participants were contacted during the first prenatal visit to one of twelve sexual and reproductive health care services (ASSIR) of the Catalan Institute of Health (ICS) in Tarragona, Spain. The pregnant women were monitored from the first trimester (12th gestational week) to the third trimester (36th gestational week) of pregnancy. The inclusion criteria were: healthy woman older than 18 years of age, pregnancy at ≤12 weeks, ability to understand the local and official state languages and the characteristics of the study, and signing the informed consent form. The women were excluded if they fulfilled any of the following exclusion criteria: multiple pregnancy, having taken iron supplements during the months previous to week 12 of pregnancy, hypersensitivity to egg protein, previous severe disease (immunosuppression) or any chronic disease which could affect their nutritional development (cancer, diabetes, malabsorption, or liver disease).

The present study is a secondary blood sample analysis of a randomized controlled trial of iron supplementation during pregnancy, called the ECLIPSES study. Finally, in the ECLIPSES study, 791 women were recruited at week 12 of pregnancy, and 534 women completed the data on week 36 of pregnancy. The causes of dropout included leaving voluntarily, miscarriage, the emergence of exclusion criteria during pregnancy (such as serious illness that could affect the nutritional status, for instance cancer, diabetes, malabsorption or liver disease) and lost to follow-up. More information can be found in the previous paper [23].

This study was approved by the Clinical Research Ethics Committee of the Jordi Gol Institute for Primary Care Research (IDIAP) and the Pere Virgili Institute for Health Research (IISPV). All participants who agreed to participate signed the informed consent form.

### Data collected

Midwives collected the participants’ medical and obstetric history, socioeconomic information, lifestyle habits and anthropometric measurements in the first trimester of pregnancy. The medical and socioeconomic data included maternal age, ethnicity, education level (primary, secondary, or university studies), smoking habits and planned pregnancy, clinical and obstetric history. The socioeconomic level was classified as low, middle or high according to the Catalan classification of occupations (CCO-2011) [24]. Anthropometric measurements were obtained such as maternal height (cm) and weight (Kg), and BMI was calculated. The BMI was categorized as normal weight (BMI < 25 kg/m^2^) or excess weight (BMI ≥25 kg/m^2^) according to the World Health Organization (WHO) criteria [25].

#### Blood sampling and determination of the fatty acid profile in plasma

Blood samples were collected at the first and third trimesters of pregnancy. They were collected in 1 tube of 7.5 ml without anticoagulant and were not mixed for 30 min at room temperature to allow coagulation. The serum was centrifuged and distributed in aliquots of 500 μl and stored at − 80 °C. The samples were stored in the BioBank. At the end of the clinical study, samples were thawed and processed simultaneously in order to minimize inter-batch variation [22].

FA such as saturated, mono- and polyunsaturated FA were analysed. A combination of gas chromatography–mass spectrometry (GC-MS) was used to analyse FA using 7890A GC equipment coupled to QqQ 7000 Series® (Agilent Technologies Inc., Santa Clara, USA) after their derivatization to methyl ester (FAMEs) due to their higher volatility [26]. A volume of 50 μl of plasma samples was blended with internal standard (IS) solution (Myristic d-27 acid, Merck KGaA, Darmstadt, Germany), chloroform and methanolic hydrochloric acid and incubated at 80 °C during 2 hours. Then, FAMEs were extracted by liquid-liquid extraction by means of hexane and then injected in the GC-MS system. Chromatographic analysis was carried out according to David et al. [26] to determine the 37 FAMEs included in the Food Industry FAME Mix (Restek Corporation, Pennsylvania, USA). FAMEs were split up in a high-polarity column (100 m × 250 μm × 0.25 μm) (HP-88 column, Agilent Technologies Inc., USA) with a temperature program between 140 and 240 °C at 1 mL/min by a carrier gas (helium). Ionization was performed by electronic impart (70 eV) and the mass analyser operated on Selected Ion Monitoring mode (SIM). The processing of the FA sample was described in the previous paper [27]. The FA identified were: SFA, lauric acid, myristic acid, palmitic acid and stearic acid (the sum total of these was calculated to obtain total SFA); MUFA, palmitoleic acid and oleic acid (the sum of these FA was calculated to obtain total MUFA); n-6 PUFA, linoleic acid (LA), dihomo-γ-linolenic acid and AA (the sum total of these was calculated to obtain total n-6 PUFA); n-3 PUFA, EPA and DHA (the sum of these FA was calculated to obtain total n-3 PUFA). Moreover, total long chain fatty acids was calculated as total SFA + total MUFA + total n-6 PUFA + total n-3 MUFA.

### Statistical analysis

The ability of the study sample to detect differences in FA concentration between the first and third trimesters of pregnancy was calculated using the data from this study in accordance with the following parameters: an alpha risk of 0.05 and a beta risk of 0.20 in a bilateral contrast to paired samples. For total SFA, 444 participants were sufficient to detect a difference equal to or greater than 612 μmol/L considering a standard deviation of 4601.2 μmol/L (mean of 5860,5 μmol/L). For total MUFA, 440 participants were sufficient to detect a difference equal to or greater than 198 μmol/L considering a standard deviation of 1481.8 μmol/L (mean of 1294.7 μmol/L). For total n-6 PUFA, 443 participants were sufficient to detect a difference equal to or greater than 403 μmol/L considering a standard deviation of 3026.1 μmol/L (mean of 2844.3 μmol/L). For total n-3 PUFA, 454 participants were sufficient to detect a difference equal to or greater than 13.5 μmol/L considering a standard deviation of 102.7 μmol/L (mean of − 16.5 μmol/L). Therefore, our sample showed enough statistical power to detect differences.

For the FA concentration, outlier values were identified by z-score analysis [28], considering an absolute value of ±3.29 as the standard value to detect outliers when the sample size is > 100. Thus, an outlier case was considered when the z-score was above ±3.29 or below ±3.29 [29]. The results were presented as mean ± standard deviation (SD) or percentage. The means between groups (the first and third trimesters of pregnancy) were compared by the paired student’s t-test. The Pearson correlation was used to assess the association between each FA concentration total at the first and third trimesters. The percentage of variation from the first to third trimester was calculated for each individual FA with the following equation: (final value - initial value)/initial value*100. Statistical analyses were run by SPSS version 25.0 for Windows (SPSS, Chicago, IL, USA). The significance level was at *p*-value < 0.05.

## Results

### Participants’ characteristics

The biochemical profiles of the FA in the first and third trimesters were analysed in 479 pregnant women. The baseline characteristics of pregnant women are shown in Table 1. The maternal age was 30.6 ± 5.01 years, and up to 80% were Caucasian. About 38.3% of women had a medium educational level and 69.6% were middle-class. Likewise, 25.3 and 12.5% of the pregnant women in the sample were overweight or obese, respectively, and 15.3% of participants reported that they smoked at the beginning of pregnancy. Some variables of baseline characteristics showed missing values, which range from 0.2 to 8.7%.

**Table 1:** Baseline characteristics of pregnant women in the first trimester

General characteristics	Mean ± SD^a^ or % (*n* = 479)
Maternal age (years)^a^	30.6 ± 5.01
Parity (yes %)	58.8
Planned pregnancy (yes %)	80.7
Maternal ethnic origin (%)	
Caucasian	81.9
Asian	0.7
Arab	7.8
Black	2.1
Latin American	7.6
Maternal educational level (%)	
Low (primary or less)	30.1
Medium (secondary)	38.3
High (university or more)	31.6
Socioeconomic status (%)	
Low	13.5
Middle	69.6
High	16.9
BMI (kg/m^2^) at first trimester (%)	
< 25	62.2
25–30	25.3
≥ 30	12.5
Smoking status (%)	
Smoker	15.3
Non-Smoker or Ex-Smoker	84.7

^a^ Mean ± standard deviation. *BMI* body mass index

The FA detected in maternal serum and their concentration (μmol/L) during the first and third trimesters of pregnancy are shown in Table 2. From the first trimester to the third trimester of pregnancy there was a significant increase in total SFA (and their individual FA) and total MUFA (as well as palmitoleic acid and oleic acid) (*p* < 0.001). The percentage of variation of total SFA and total MUFA was 101.02 ± 93.05 and 184.93 ± 154.49, respectively. In addition, a significant increase in total n-6 PUFA in the third trimester (*p* < 0.0001) was observed when it was compared to the maternal FA concentration in the first trimester of pregnancy, with a percentage of 77.85 ± 85.91 of variation. Nevertheless, the percentage of variation of AA was − 6.33 ± 34.88 with a significant decrease in its concentration from the first to third trimesters. Regarding n-3 PUFA, the EPA concentration decreased during gestation (*p* < 0.001) with a percentage of variation of − 14.93 ± 68.21. A statistically non-significant result was observed in the DHA concentration (*p* = 0.357) between the first and third trimesters of pregnancy.

**Table 2:** Fatty acid concentration of maternal serum during pregnancy period from the first trimester to third trimester

Fatty acids (μmol/L)		First Trimester (T1) (Mean ± SD)	Third Trimester (T3) (Mean ± SD)	*p-*value between^(a-b)^	*% of variation from T1 to T3* (Mean ± SD)
SFA					
Lauric acid (C12:0)	*n* = 444	40.14 ± 10.61	60.19 ± 28.26	< 0.0001	56.33 ± 76.07
Myristic acid (C14:0)	*n* = 450	118.16 ± 49.07	207.80 ± 80.31	< 0.0001	96.32 ± 94.19
Palmitic acid (C16:0)	*n* = 443	2904.40 ± 1403.47	8511.32 ± 4293.76	< 0.0001	234.69 ± 195.97
Stearic acid (C18:0)	*n* = 451	690.31 ± 208.05	808.08 ± 202.10	< 0.0001	25.99 ± 44.26
Total SFA	*n* = 444	3765.31 ± 1614.17	9625.65 ± 4574.33	< 0.0001	184.93 ± 154.49
MUFA					
Palmitoleic acid (C16:1n-7)	*n* = 444	186.37 ± 45.88	256.66 ± 89.61	< 0.0001	42.94 ± 54.20
Oleic acid (C18:1n-9)	*n* = 441	1444.74 ± 461.90	2843.85 ± 1251.92	< 0.0001	108.61 ± 100.53
Total MUFA	*n* = 442	1634.54 ± 500.21	3116.39 ± 1330.86	< 0.0001	101.02 ± 93.05
n-6 PUFA					
Linoleic acid (LA) (C18:2n-6)	*n* = 446	3355.67 ± 1230.19	6321.14 ± 2786.02	< 0.0001	107.35 ± 111.69
Dihomo-γ-linolenic acid (DHGLA) (C20:3n-6)	*n* = 450	229.99 ± 90.64	246.20 ± 85.27	0.001	19.38 ± 53.87
Arachidonic acid (AA) (C20:4n-6)	*n* = 451	830.82 ± 276.15	722.63 ± 220.05	< 0.0001	−6.33 ± 34.88
Total n-6 PUFA	*n* = 444	4433.77 ± 1469.64	7278.06 ± 2919.04	< 0.0001	77.85 ± 85.91
n-3 PUFA					
Eicosapentaenoic acid (EPA) (C20:5n-3)	*n* = 446	35.03 ± 23.95	23.88 ± 16.93	< 0.0001	−14.93 ± 68.21
Docosahexaenoic acid (DHA) (C22:6n-3)	*n* = 456	240.28 ± 73.47	236.60 ± 71.77	0.357	4.89 ± 37.7
Total n-3 PUFA	*n* = 454	276.98 ± 94.07	260.52 ± 84.33	0.001	1.21 ± 38.34
Total LCFA	*n* = 442	10,073.15 ± 3349.07	20,480.82 ± 8335.23	< 0.0001	118.63 ± 103.14

Fatty acid concentration (μmol/L) of maternal serum and % of variation from the first to third trimester are presented as mean ± standard deviation (SD). SFA, saturated fatty acids; MUFA, monounsaturated fatty acids; n-6 PUFA, omega-6 polyunsaturated fatty acids; n-3 PUFA, omega-3 polyunsaturated fatty acids. Total LCFA, long chain fatty acids = total SFA + total MUFA + total n-6 PUFA + total n-3 MUFA

Moreover, as the original study was a clinical trial with iron supplementation, we analysed whether the study group had any effect on the FA concentration. There were no significant differences in FA concentrations between the study groups in the first trimester or in the second trimester.

**Fig. 1:**
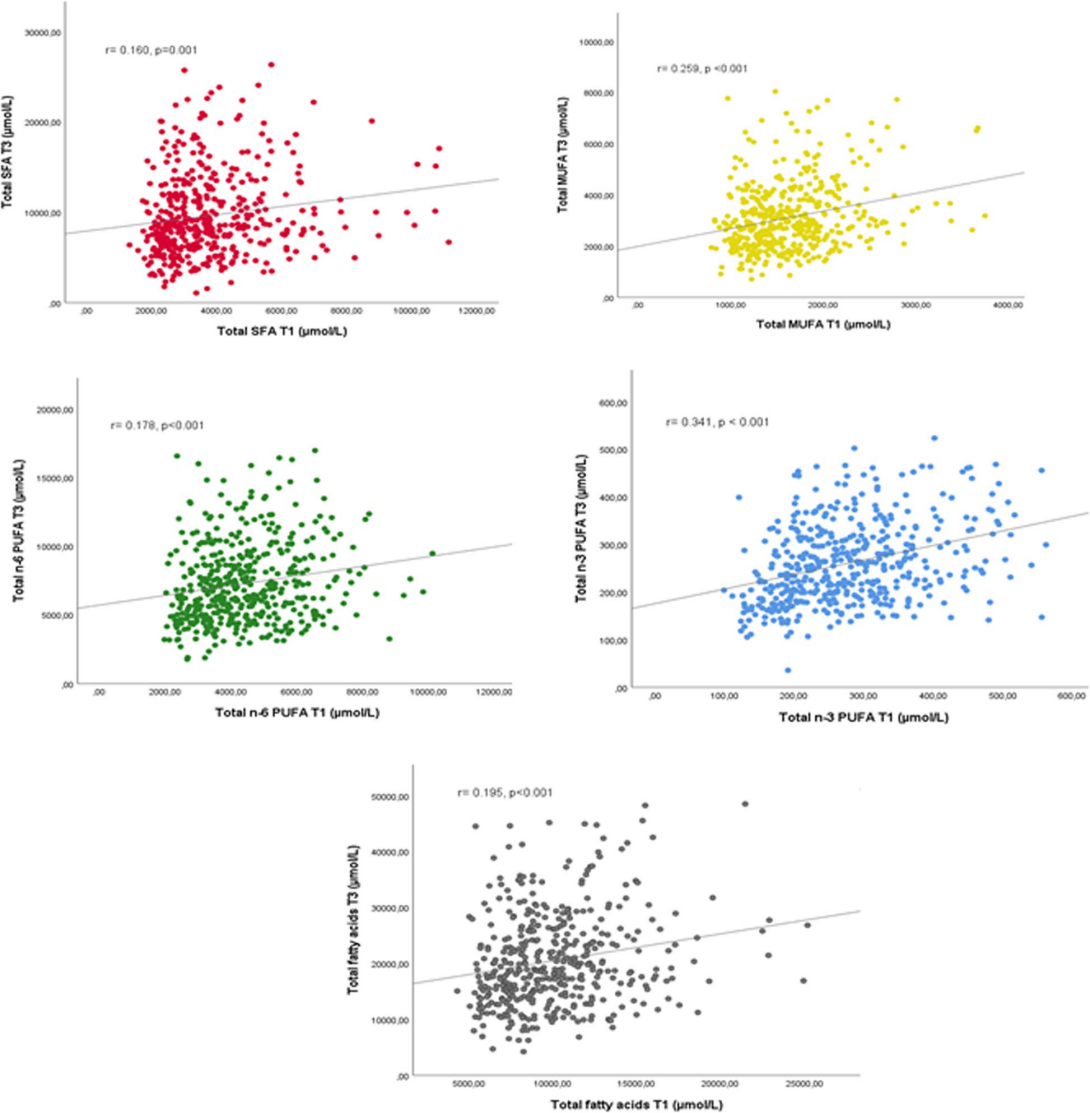
Correlation of serum concentration of total SFA, MUFA, n-6 PUFA, n-3 PUFA and total fatty acids between the first trimester (T1) and third trimester (T3). SFA, saturated fatty acids; MUFA, monounsaturated fatty acids; n-6 PUFA, omega-6 polyunsaturated fatty acid; n-3 PUFA, omega-3 polyunsaturated fatty acid. Total fatty acids = total SFA + total MUFA + total n-6 PUFA + total n-3 MUFA

Moreover, Fig. 1 depicts the correlation of serum concentration of total SFA, MUFA, n-6 PUFA, n-3 PUFA and total FA between the first trimester and third trimester. A significantly low-moderate correlation of each total FA concentration was observed from the first to third trimesters.

## Discussion

This longitudinal study describes the changes in FA during pregnancy in a larger sample of women than in previous studies and, to our knowledge, for the first time in a population from the Mediterranean area. It provides valuable data on a wide range of FA, these being total SFA and lauric acid, myristic acid, palmitic acid and stearic acid; total MUFA and palmitoleic acid and oleic acid; total n-6 PUFA and linoleic acid, dihomo-γ-linolenic acid and AA; n-3 PUFA and EPA and total DHA. The data on these FA were determined using high-quality methods and evaluated at two moments of gestation (first and third trimester). This has made it possible to describe changes to the different types of FA during pregnancy. The concentrations of SFA, MUFA and PUFA n-6 increased from the first to the third trimester of gestation, except for AA, the concentrations of which decrease. In contrast, n-3 PUFA and EPA concentrations decreased during pregnancy, while DHA concentrations remained stable. It appears that essential fatty acids DHA and AA, which have a preferential placental transfer to the foetus, do not evolve in the same way as other FA during pregnancy.

There are few research projects that have analysed SFA and MUFA serum concentrations throughout pregnancy, conducted with Brazilian and Dutch pregnant woman [17–19, 21]. Our results are consistent with their findings, as they also found an increase in SFA and MUFA during pregnancy. This increase could be due to an increase in biosynthesis to cover requirements, since the rise in serum concentration may not be related to the small variation in the mothers’ dietary intake [30, 31], as our research group found in this sample of healthy pregnant women in relation to their intake during pregnancy [32]. The SFA may play a key role in supporting foetal membrane growth, as the SFA bio-magnification process could help to satisfy AA demands in foetal circulation and DHA in the brain [33]. However, the crucial role played by SFA has yet to be determined. An adequate concentration of MUFA is also fundamental during pregnancy because a non-optimal level has been associated with a high risk of preterm delivery and low weight at birth [33]. Oleic acid concentration could be a biomarker for a lack of essential fatty acid in the mother’s diet, as has been shown in animal models [33]. In addition, it has been suggested that the consumption of monounsaturated fats could benefit DHA metabolism because they have a low amount of LA and thus do not interfere with endogenous conversion of α-linolenic acid (ALA) to DHA [34]. Further research is needed to analyse their role during pregnancy.

In terms of PUFA, we found that n-6 PUFA concentrations increased during gestation, whereas essential fatty acid (EPA, DHA, AA) concentrations decreased or remained stable. Our results are partially in concordance with the literature. For instance, Zhao et al. [20] observed that Canadian pregnant women without diabetes showed higher serum concentrations of all fatty acids except EPA at 35–37 weeks of pregnancy than at 24–28 weeks. A systematic review found that levels of absolute concentrations of DHA increased and EPA did not change [16]. Pinto et al. [17], in a study involving Brazilian pregnant women, showed that from the first to second trimesters the serum FA concentrations, EPA + DHA, increased greatly, but a slight increase in DHA and a decrease in EPA (although not statistically significant) were observed in the third trimester. However, our study found no significant variation in DHA from the first to third trimesters and observed a significant decrease of around 15% in EPA concentrations. It may be that the DHA synthesis from EPA was more efficient than from ALA [35, 36] and that DHA concentrations could be regulated by a different biosynthesis or mobilization mechanism [35–38]. In fact, n-6 and n-3 PUFA families compete for metabolism by desaturation enzymes, and therefore a Western or industrialized diet rich in vegetable oils that are high in LA would inhibit the synthesis of n-3 PUFA by ALA. In addition, AA is an essential fatty acid that is preferential for transfer to the foetus along with DHA [1]. Our findings show that AA concentrations also decrease by around 8% during pregnancy. Although LA serum concentrations increased, it does not appear that it was to be converted into AA, which may suggest that enzymatic activity of delta 5 desaturase could be reduced. Other researchers have suggested that LA may compete with other fatty acids, including AA, for acylation [39, 40]. In agreement with our results, Stewart et al. [38] found no differences in the AA concentrations of erythrocytes; however, other researchers have found an increase in AA serum concentrations during pregnancy [16, 17, 20]. The reasons for the different findings are unclear. It should be noted that the systematic review by Wilson et al. [16] included only three studies that assessed serum concentrations of FA, one from Brazil and two from the Netherlands, whose populations have different dietary patterns with a high level of heterogeneity between them. It is possible that the differences could be due to when the measurements were taken, to sample size, the fatty acid assay protocol or the study populations. More research is needed to clarify how essential fatty acids change over the course of the entire pregnancy.

Furthermore, our study is a secondary analysis of a clinical trial of iron supplementation. In this sense, a physiological relationship between iron and fatty acids is hypostatized since, for instance, ferritin may incorporate FA in its structure in addition to iron [41, 42]. However, our results did not show that the iron dosage of the supplementation had any effect on FA concentration.

To our knowledge this is the first study of the changes in maternal serum FA during pregnancy in a Mediterranean region. According our findings, circulating FA – specifically SFA, MUFA and n-6 PUFA – increases throughout pregnancy. This could be explained by a synthesis mechanism or mobilization of the maternal fat store, since the concentrations may not be related to dietary intake. Given this situation, we could consider that the increase in SFA, MUFA and n-6 PUFA serum concentration during pregnancy seems to indicate that the metabolic mechanisms involved may offer an optimal nutritional availability to meet the increased demands of these FA due to the increased growth and development of the foetus at the end of gestation. Nevertheless, essential fatty acid concentrations, which are preferential for transfer to the placenta, may be more complexly regulated. During early pregnancy the PUFA derived from the diet are stored in maternal adipose tissue. Indeed, n-3 PUFA serum concentration has been related to food consumption in pregnant woman [8, 10, 17, 27] insofar as it is transferred from intake to maternal fat stores. The amount of n-3 PUFA stored in the maternal adipose tissue could vary depending on the habitual dietary intake of n-3 fatty acids. However, metabolic and physiological changes occur during pregnancy, and these involve the complex synchronization of maternal, placental and foetal fat metabolism to ensure a continuous supply of n-3 and n-6 PUFA to the foetus [35]. Some mechanisms could be related to the mobilization of maternal fat stores, an increase in the elongation and desaturation of FA regulated by oestrogen, the differential placental uptake of FA or the rate of DHA conversion synchronized according to the period of maximal foetal demand [35–38]. The transfer of n-3 and n-6 PUFA to the foetus increases from 20 weeks of gestation and reaches a peak in the last trimester of pregnancy with a high maternal transfer of DHA and AA to ensure foetal demand [1, 35]. Overall, this decrease or non-modification of essential fatty acids during pregnancy, closely related to their intake, indicates that there are specific physiological mechanisms, such as preferential FA transfer between mother and foetus, so as to ensure high foetal demands are met [35]. This might put the mother at risk of deficiency when her intake is insufficient. Indeed, it has been found that low n-3 PUFA status and imbalance between n-6 and n-3 PUFA in early pregnancy increase the risk of postpartum depression after delivery [10, 14]. This could lead us to consider that the mechanisms involved in its storage in maternal fat, together with other possible complex mechanisms of metabolic synchronization of maternal, placental and foetal fat could be insufficient to guarantee the FA needs in situations of low contributions. In general, for all FA, further testing of normal FA levels during pregnancy would be necessary for better evaluations and follow-ups. In addition, more research is needed on the metabolic mechanisms of FA during pregnancy.

Given that essential fatty acid concentrations and their balance depend on both biological mechanisms and dietary supply, an optimal dietary intake is needed to ensure proper functioning of biological mechanisms so as to facilitate correct foetal growth and development. For this reason, we believe that the pregnancy control reviews carried out by obstetricians and health professionals should include dietary advice on foods rich in these FA, verify consumption of these foods and thus FA, and determine the serum concentrations of essential FA. There is some evidence that supports the benefits of an adequate intake of FA. Fish and seafood consumption during pregnancy has been reported to be beneficial for offspring neurocognitive development [43, 44]. Several other research projects have associated an inadequate balance of n-3 and n-6 PUFA profiles during pregnancy with deleterious consequences for the child, such as neurodevelopment disorder [43, 6], immune system disease [45, 46] and cardiometabolic risk and adiposity [47, 48]. In addition, AA is a precursor of prostaglandin and other metabolites, which play a key role in the late pregnancy period and delivery [49]. Nevertheless, an excessive activation of inflammatory mediators during pregnancy might lead to deleterious effects, such as gestational diabetes, preeclampsia or pre-term delivery [15].

The main strength of our study is its longitudinal design, which enabled us to analyse variations in serum FA concentrations throughout pregnancy. Another strength is the bigger sample size compared to other research [17–21]. However, several limitations should be considered**.** Although the sample is of voluntary pregnant women from a randomized clinical trial with a control group, the sample avoids the selective survival error by selecting women from each area of the primary health care ASSIR service specific for pregnant women, from an entire region. This provided a good representation of the socioeconomic and educational levels of the sample, among other aspects. In addition, exhaustive inclusion and exclusion criteria were fulfilled, previously designed and agreed on with the ASSIR service, thus guaranteeing a sample of healthy pregnant adult women. Incentives were given to minimize the loss of women in the pregnancy follow-up, consisting of follow-up visits interspersed with study visits, economic incentives for midwives who carried out these visits, and informational incentives for mothers about their health status and that of their baby. In the design phase of the study, all procedures, masking and quality control were systematized and monitored by a group external to the research group. Nevertheless, our findings may not be extended to all populations as ethnicity, nutritional habits and lifestyle may impact on the fatty acid levels. In the assessment of FA, it was not possible to assess ALA, one of the main n-3 PUFA, in our sample. Furthermore, due to the lack of reference values, our study was unable to apply a cut-off value regarding the percentage of the pregnant population that is above or below the optimal level. Therefore, our findings indicate that it is necessary to establish reference values according to the variation of FA in each trimester. Moreover, taking into account that our results show a decrease in essential fatty acids at the end of pregnancy due to the demands of the foetus, health and nutrition counselling provided by obstetricians and health professionals could add that mothers consume foods rich in FA from the beginning of the pregnancy to prevent levels from dropping excessively at the end of gestation. In addition, clinical guidelines during pregnancy could include the assessment of FA in each trimester, so that professionals could identify those women who are at risk and recommend specific treatment or advice if necessary.

In conclusion, the circulating serum concentration of SFA, MUFA and n-6 PUFA increases during pregnancy, whereas essential fatty acids such as AA and EPA decrease and DHA remains unchanged. Therefore, the increase in SFA, MUFA and n-6 PUFA serum concentration during pregnancy, which is related to specific metabolic mechanisms during pregnancy, seems to offer an adequate availability of these FA for increased foetal growth and development at the end of pregnancy. However, the decrease or non-increase in essential fatty acids during pregnancy may indicate that there are specific physiological mechanisms that might put the mother at risk of deficiency when her intake is insufficient. More research is necessary to analyse the role of FA during pregnancy.

## Data Availability

The datasets used and/or analysed during the current study are only available from the corresponding author on reasonable request.

## Abbreviations

FA: Fatty acids
SFA: Saturated fatty acids
MUFA: Monounsaturated fatty acids
PUFA: Polyunsaturated fatty acids
n-6 PUFA: Omega-6 polyunsaturated fatty acids
n-3 PUFA: Omega-3 polyunsaturated fatty acids
DHA: Docosahexaenoic acid
EPA: Eicosapentaenoic acid
AA: Arachidonic acid
LA: Linoleic acid
ALA: α-linolenic acid
